# Diesel engine exhaust and lung cancer risks – evaluation of the meta-analysis by Vermeulen et al. 2014

**DOI:** 10.1186/s12995-015-0073-6

**Published:** 2015-08-12

**Authors:** Peter Morfeld, Michael Spallek

**Affiliations:** Institute and Policlinic for Occupational Medicine, Environmental Medicine and Prevention Research of Cologne University, Kerpener Str. 62, 50937 Köln, Germany; Institute for Occupational Epidemiology and Risk Assessment (IERA) of Evonik Industries AG, Rellinghauser Str. 1-11, D-45128 Essen, Germany; Institute for Occupational, Social and Environmental Medicine of the Goethe University Frankfurt/Main, Theodor-Stern-Kai 7, 60590 Frankfurt am Main, Germany; European Research Group on Environment and Health in the Transport Sector (EUGT e.V.), Fritschestr. 35, 10627 Berlin, Germany

**Keywords:** Diesel, DEE, Lung cancer, Epidemiology, Meta-analysis

## Abstract

**Background:**

Vermeulen et al. 2014 published a meta-regression analysis of three relevant epidemiological US studies (Steenland et al. 1998, Garshick et al. 2012, Silverman et al. 2012) that estimated the association between occupational diesel engine exhaust (DEE) exposure and lung cancer mortality. The DEE exposure was measured as cumulative exposure to estimated respirable elemental carbon in μg/m^3^-years. Vermeulen et al. 2014 found a statistically significant dose–response association and described elevated lung cancer risks even at very low exposures.

**Methods:**

We performed an extended re-analysis using different modelling approaches (fixed and random effects regression analyses, Greenland/Longnecker method) and explored the impact of varying input data (modified coefficients of Garshick et al. 2012, results from Crump et al. 2015 replacing Silverman et al. 2012, modified analysis of Moehner et al. 2013).

**Results:**

We reproduced the individual and main meta-analytical results of Vermeulen et al. 2014. However, our analysis demonstrated a heterogeneity of the baseline relative risk levels between the three studies. This heterogeneity was reduced after the coefficients of Garshick et al. 2012 were modified while the dose coefficient dropped by an order of magnitude for this study and was far from being significant (*P* = 0.6). A (non-significant) threshold estimate for the cumulative DEE exposure was found at 150 μg/m^3^-years when extending the meta-analyses of the three studies by hockey-stick regression modelling (including the modified coefficients for Garshick et al. 2012). The data used by Vermeulen and colleagues led to the highest relative risk estimate across all sensitivity analyses performed. The lowest relative risk estimate was found after exclusion of the explorative study by Steenland et al. 1998 in a meta-regression analysis of Garshick et al. 2012 (modified), Silverman et al. 2012 (modified according to Crump et al. 2015) and Möhner et al. 2013. The meta-coefficient was estimated to be about 10–20 % of the main effect estimate in Vermeulen et al. 2014 in this analysis.

**Conclusions:**

The findings of Vermeulen et al. 2014 should not be used without reservations in any risk assessments. This is particularly true for the low end of the exposure scale.

## Background

Vermeulen et al. [1] published a meta-regression analysis of three major epidemiological US studies [2–4] which investigated the association between diesel engine exhaust (DEE) exposure – based on the cumulative exposure to respirable elemental carbon (REC) in μg/m^3^-years – and lung cancer mortality. The authors reported that some of them worked as members of the IARC Working Group (see [5]), which produced a review of the carcinogenic effect of DEE (hazard assessment) and classified DEE as a human carcinogen (IARC Group 1) in 2012. With this follow-up analysis, Vermeulen et al. [1] aimed to continue these considerations and contribute to the quantitative risk assessment based on the major studies. From the data of the three studies, the authors’ main result was a common exposure-risk curve without a threshold and concluded: “We estimated a lnRR of 0.00098 (95 % CI: 0.00055, 0.0014) for lung cancer mortality with each 1-μg/m^3^-year increase in cumulative EC based on a linear meta-regression model. Corresponding lnRRs for the individual studies ranged from 0.00061 to 0.0012. Estimated numbers of excess lung cancer deaths through 80 years of age for lifetime occupational exposures of 1, 10, and 25 μg/m^3^ EC were 17, 200, and 689 per 10,000, respectively. For lifetime environmental exposure to 0.8 μg/m^3^ EC, we estimated 21 excess lung cancer deaths per 10,000. Our estimates suggest that stringent occupational and environmental standards for DEE should be set.” So the paper describes elevated risks of cancer even at very low DEE exposures. As a result, the Vermeulen et al. [1] paper will play an important role in the ongoing DEE limit value discussions. The US National Institute for Occupational Health (NIOSH) has already attempted to derive estimates from this using its standard procedure (excess case calculation) [6]: Based on Vermeulen et al. [1], Dr. Park calculated an estimate for the 8 h DEE threshold at the workplace of 0.59 μg/m^3^.

In contrast, the EU’s ACSH [7] recommended a limit value of 100 μg/m^3^ for DEEE (diesel engine exhaust emissions), measured as elemental carbon. This recommendation is not health-based but reflects mainly socio-economic considerations. Cherrie et al. [8] concluded that only 2 % of workers exposed to DEEE are estimated to be exposed above this level in the EU. In addition, the authors wrote: “There is a case for introducing an OEL [occupational exposure limit] for DEE particulate, but the OEL would need to be much lower than the typical European OEL that we tested (0.1 mg/m^3^)”. The proposal derived by Park [6] using Vermeulen et al. [1] is such a proposal that is much lower than the recommendation of the EU’s ACSH [7]. A critical analysis of the meta-regression approach by Vermeulen et al. [1] is indicated to understand whether the Vermeulen et al. analysis presents evidence to support such a low limit value, far-off the 100 μg/m^3^ limit value recommendation of ACSH [7].

Throughout this paper, we use “dose” as an abbreviation for “cumulative exposure to respirable elemental carbon (REC) in μg/m^3^-years”.

## Material

### Vermeulen et al. 2014: input data for primary analysis

The meta-regression analysis of Vermeulen et al. [1] included three epidemiological US studies (Steenland et al. 1998 [2], Garshick et al. 2012 [3], Silverman et al. 2012 [4]) that estimated the association between occupational DEE exposure, measured as cumulative REC exposure in μg/m^3^-years (dose), and lung cancer mortality.

Steenland et al. [2] is a nested case–control study on workers in the US trucking industry (994 lung cancer deaths and 1085 controls). The dose values were lagged 5 years when calculating the odds ratios (OR). Lagging is an evaluation technique which discards the exposure data of the last years (in this case the last 5 years) to take cancer latency phenomena into account [9]. This is the oldest study in the analysis, as it is based on the data from the case–control study by Steenland et al. [10]: “all cases and controls had died in 1982–1983.” The measurements for elemental carbon by Zaebst et al. [11], which were not collected until 1990, were used as the basis for exposure estimation for the period from 1949 to 1983. Accordingly, the measurements were taken approx. 8 years after the death of the persons in the study, and therefore even later after the end of their exposure phase. Steenland et al. [2] attempted to extrapolate these data back “dependent on very broad assumptions”. The authors evaluated this key limitation of their research as follows: “Our results should be regarded with appropriate caution because our exposure estimates are based on broad assumptions rather than actual measurements” and they noted the following in the abstract: “Our results depend on estimates about unknown past exposures, and should be viewed as exploratory.”

Garshick et al. [3] is a cohort study independent of this on the US trucking industry (31,135 male employees, 779 lung cancer deaths). Date of death and cause-specific mortality was obtained from 1985 through 2000. Historical trends in ambient terminal REC were estimated based on historical trends in the coefficient of haze available for 1971 through 2000, a measurement of particulate matter based on optical density, assumed to be predictive of ambient REC. Vermeulen et al. [1] used this cohort data after excluding mechanics, and also used dose values lagged by 5 years. Those risk estimates (hazard ratios) from the Garshick study were used which were adjusted for duration of exposure.

Silverman et al. [4] is the case–control study of the US DEMS (DEMS = Diesel Exhaust in Miners Study). The underlying cohort [12] totalled 12,315 miners from 8 non-metal mining operations and included both surface and underground workers (no ore or coal mining). The case–control study included 198 lung cancer deaths and 562 controls based on mortality follow-up through December 31, 1997. Unlike the other two studies, the authors used a lag of 15 years to calculate the odds ratios. The exposures to REC were estimated in a complicated manner based on measurements of carbon monoxide (CO) and REC made in 1998–2000 and estimates of diesel equipment horsepower used through 1997 and mine ventilation.

The supplement to Vermeulen et al. [1] contains most of the input data to the Vermeulen meta-analyses (mean dose estimate for each dose category of the studies incorporated and the corresponding relative risk RR with a 95 % confidence interval). The data were extracted and transferred to a Stata file. Gaps were filled, where the original individual study publications contained the missing information.

Table 1 shows the input data as incorporated in the primary analysis by Vermeulen et al. [1]. The three studies included [2–4] reported cumulative exposure to DEE as respirable elemental carbon (dose) with relative risks and the corresponding 95 % confidence intervals. While the dose is reported using different categorizations, all three papers use μg/m^3^-years as the dose unit. Table 1 lists ORs and HRs uniformly as relative risks.

**Table 1:** Input data on the primary analysis in Vermeulen et al. [1]

Study	lag/a	Exposure category	Average dose	Lower dose	Upper dose	RR	95 % CI		Number of persons	Number of cases
							Lower	Upper		
Steenland et al. 1998	5	Reference	0.0	0.0	0.0	1.00	1.00	1.00		
	5	Cat 1	84.5	0.0	<169.0	1.08	0.72	1.63		
	5	Cat 2	231.0	169.0	257.0	1.10	0.74	1.65		
	5	Cat 3	294.0	257.0	331.0	1.36	0.90	2.04		
	5	Cat 4	551.7	≥331.0		1.64	1.09	2.49		
Garshick et al. 2012^a^	5	Reference	15.5	0.0	<30.9	1.00	1.00	1.00	105513	122
	5	Cat 1	51.3	30.9	71.7	1.31	1.01	1.71	104909	191
	5	Cat 2	111.0	71.7	150.3	1.38	1.02	1.87	102496	202
	5	Cat 3	250.5	≥150.3		1.48	1.05	2.10	87397	226
Silverman et al. 2012	15	Reference	1.5	0.0	<3.0	1.00	1.00	1.00	207	49
	15	Cat 1	37.5	3.0	72.0	0.74	0.40	1.38	278	50
	15	Cat 2	204.0	72.0	536.0	1.54	0.74	3.20	206	49
	15	Cat 3	1036.0	≥536.0		2.83	1.28	6.26	173	50

Dose refers to the cumulative exposure to DEE in μg/m^3^-years. For every category (Reference, Cat 1, Cat 2, Cat 3 and Cat 4), “averages” and the lower and upper limits of the dose are specified. Estimates of the relative risk RR with 95 % confidence interval (95 % CI) per category are given. Study size and number of lung cancer deaths are reported

^a^Garshick et al. [3] do not include mechanics as an employee group, and the risk estimates are adjusted for duration of exposure. For Garshick et al. [3], the number of person years is stated instead of the number of persons, as it is a cohort study. Steenland et al. [2] specify neither the number of persons nor the number of cases per exposure category

For a more detailed description of the studies incorporated, we refer to Vermeulen et al. [1] and the original publications.

### Corrected estimates: Garshick et al. 2012 (modified)

All analyses in Vermeulen et al. [1] applied the risk estimates (hazard ratios) from the Garshick study that were additionally adjusted for duration of exposure. An important aspect in this context is evident from the Letter to the Editor by Morfeld [13] (including the authors’ answer) on adjustment errors in the coefficients used by Vermeulen et al. [1]. Morfeld criticised that the cumulative exposure was adjusted for duration of exposure, it already contains per definition. Thus, the risk coefficient does not estimate the effect of cumulative exposure, but that of a concentration (although this is not an optimal approach to estimating the concentration effect). The authors responded to the criticism as follows: “Morfeld suggests that adjusting cumulative exposure by duration of employment time reduces cumulative exposure to an estimate of long-term average concentration. We agree that if exposure in our workers was relatively constant, cumulative exposure would be the simple product of duration and average exposure. However, exposure varies considerably over time and between and within jobs.” We emphasize that this note does not justify the procedure, as the data was evaluated using time-dependent methods in the Cox analyses performed. So the following applies for every point in time and in every person: cumulative exposure = duration of exposure × average concentration. Adjusting for duration of exposure changes the analysis such that time-dependent average concentration is analysed, not the cumulative exposure. The authors justify the exposure duration adjustment they made in spite of this by claiming that it controls for the healthy worker survivor effect. However, other methods are required for this [9].

Neither of the other studies [2, 4] made adjustments of this type to the cumulative exposure. Another aspect of the Garshick study, which also affects the model specification, is covered in the discussion section.

Garshick et al. [3] offered coefficients of cumulative exposure without adjustment for duration of exposure. In order to give the coefficients their usual meaning, and to facilitate comparison with the other two studies, these coefficients were also included in this analysis. The data are shown in Table 2 and will be referred to as Garshick et al. [3] (modified) below.

**Table 2:** Results from Garshick et al. [3] without adjustment for duration of exposure, referred to as Garshick et al. [3] (modified)

Exposure category	Average dose	Lower dose	Upper dose	RR	95 % CI		Person years	Number of cases
					Lower	Upper		
Reference	15.5	0.0	<30.9	1.00	1.00	1.00	105513	122
Cat 1	51.3	30.9	71.7	1.18	0.92	1.52	104909	191
Cat 2	111.0	71.7	150.3	1.17	0.88	1.55	102496	202
Cat 3	250.5	≥150.3		1.19	0.86	1.63	87397	226

Dose refers to the cumulative exposure to DEE in μg/m^3^-years. For every category (Reference, Cat 1, Cat 2, Cat 3), averages and the lower and upper limits of the dose are specified. Odds ratio OR with 95 % confidence intervals (95 % CI) and the number of person years and the number of observed lung cancer deaths per dose category are reported

The risk estimates are not only lower than in Table 1, but also do not exhibit a positive trend with increasing exposure levels.

Vermeulen et al. [1] also performed sensitivity analyses with data deviating from Garshick et al. [3] as reported in Table 1. However, these variations only involved the lag of the exposure variables and the inclusion/exclusion of the data from mechanics. By contrast, the authors do not take the problem of the cumulative exposure coefficients incorrectly adjusted for duration of exposure into consideration.

### Re-analysis of the DEMS case/control study: Crump et al. 2015

Crump et al. [14] re-analysed the DEMS case–control study by Silverman et al. [4] and largely managed to reproduce its results: “We were able to replicate the findings reported by Silverman et al. (18) when we used the same analytical methods. This gave us confidence that we were using the same basic data set as Silverman et al.”. Crump et al. investigated the influence of covariables which Silverman et al. [4] did not include in their final models. The radon exposure underground proved to be a main confounder, a result which did not match the statements by Silverman et al. [4]. On the cumulative exposure to radon, Silverman et al. [4] wrote “estimated cumulative exposure to radon … were evaluated but not included in the final models because they had little or no impact on odds ratios (i.e., inclusion of these factors in the final models changed point estimates for diesel exposure by ≤10 %)”. Crump et al. [14] noted the following on this: “However, when we reproduced the Silverman et al. analysis, we could not verify this statement.” As a result, the present sensitivity meta-analysis only uses the estimates by Crump et al. [14] after an additional adjustment for cumulative radon exposure.

Crump et al. [14] also developed six new DEE exposure metrics, as an alternative to the estimates used in Attfield et al. [12] and Silverman et al. [4]: “We proceeded to apply six alternative REC metrics, five of which depended, as did the DEMS metrics, on extrapolations involving assumed relationships between CO and REC. A sixth REC metric, REC6, was used that did not involve any assumptions concerning the relationship between CO and REC, and was based on Adj_HP [adjusted horse power] and ventilation rates for each of the mines. Of the several REC metrics, we view REC6 as having some superior qualities because it avoids using the highly uncertain assumptions concerning the relationship between CO and REC.” Therefore, we use REC6 as a primary alternative to the Silverman exposure data.

In their supplementary evaluations for the exposure estimates, Crump and van Landingham [15] determined β = 0.3 as the best estimate in the REC ~ CO^β^ conversion model, as compared with the value of β = 0.58 according to Stewart et al. [16] or β = 1.0, which Silverman et al. [4] assumed in their analyses. The alternative exposure metric REC4 of Crump et al. [14] contains this best estimate, β = 0.3. REC4 is also directly based on the work by Crump and van Landingham [15] to estimate an alternative exposure, and takes several other aspects into consideration (see the detailed description of REC1 to REC4 in Crump et al. [14]). As a result, we also used the exposure metric REC4 as a second variation according to Crump et al. [14]. Table 3 gives an overview of the results of the re-analysis with REC4 and REC6 according to Crump et al. [14]. For the sake of clarity we emphasize that we only used results based on REC4 and REC6 after adjustment for radon exposure.

**Table 3:** Re-analysis of Silverman et al. [4] with adjustment for radon exposure (Crump et al. [14], Table 3)

Exposure category	Average dose	Lower dose	Upper dose	OR	95 % CI		Number of persons	Number of cases
					Lower	Upper		
Crump et al. [14], REC4								
Reference	0.71	0	<4.9	1	1	1	217	49
Cat 1	26.65	4.9	<70.4	0.80	0.41	1.55	266	50
Cat 2	243.43	70.4	<498.4	1.67	0.73	3.81	192	49
Cat 3	1522.10	-	≥498.4	1.50	0.54	4.17	189	50
Crump et al. [14], REC6								
Reference	0.61	0	<2.8	1	1	1	230	49
Cat 1	20.47	2.8	<50.6	1.07	0.57	2.00	247	50
Cat 2	158.27	50.6	<388.0	1.35	0.62	2.94	206	49
Cat 3	1156.89	-	≥388.0	1.43	0.52	3.94	181	50

The results are based on the exposure metrics REC4 and REC6 with a lag of 15 years (see text). Dose refers to the cumulative exposure to DEE in μg/m^3^-years. For every category (Reference, Cat 1, Cat 2, Cat 3), averages and the lower and upper limits of the dose are specified. Odds ratio OR with 95 % confidence intervals (95 % CI) and the number of persons observed and the number of lung cancer deaths per dose category are reported

### Re-categorised data: Möhner et al. 2013 (adapted)

Vermeulen et al. [1] omitted the German potash miner study of Möhner et al. [17] from their analysis, as they alleged the reference category defined was too high. Möhner et al. [17] was only used in two sensitivity analyses, however it was incorporated by Vermeulen et al. [1] either leaving the original RR estimates or correcting the risk estimates ad-hoc. Both approaches are less than ideal, which is why Vermeulen et al. [1] did not present either of the evaluations as part of their primary analysis, merely reporting on the results in the online appendix.

The German Potash miner study included 5,819 miners followed in mortality from 1970 through 2001. In 1991, exposure measurements of the concentration of total and elemental carbon (to a lesser extent) in the respirable dust fraction by coulometric analysis were undertaken. Because the mining technology and the mining equipment remained fairly stable since 1969, measurements from 1991 have been used for designing a job-exposure-matrix with three main job categories: production, maintenance, and workshop. Elemental carbon was the largest component of total carbon with a proportion of weight of about 63 %. Moreover, the two measures were highly correlated (*r* = 0.89). Cumulative exposure was determined as REC in μg/m^3^-years.

In order to incorporate the German potash miner study as informatively as possible in this project, the data had to be used at a different scale to that published. We contacted the original authors for the information required to do so (the publication does not contain all relevant categorizations of exposure, and other detailed information was missing). On request, Dr. Möhner provided additional results from the Möhner et al. [17] study by e-mail on 03/07/2014. This included case numbers and odds ratio estimates with 95 % confidence intervals for a modified categorisation, referred to as “Möhner et al. [17] (adapted)” below.

The breakdown was adapted so that the reference category only consists of 5 cases, with the result that the exposure level of the reference category is far lower than in the original analysis (a criticism in Vermeulen et al. [1]). The other three categories were chosen in such a way that the case distribution is roughly equal. Table 4 shows the results of this re-categorisation of Möhner et al. [17].

**Table 4:** Additional results for the study by Möhner et al. [17] with changed (adapted) categorisation, referred to as Möhner et al. [17] (adapted)

Exposure category	Average dose	Lower dose	Upper dose	OR	95 % CI		Number of persons	Number of cases
					Lower	Upper		
Reference	263	0	≤380	1	1	1	29	5
Cat 1	728	380	1024	0.99	0.323	3.031	126	19
Cat 2	1386	1024	1840	1.69	0.53	5.403	126	25
Cat 3	2538	>1840		1.2	0.363	3.971	127	19

Dose refers to the cumulative exposure to DEE in μg/m^3^-years. For every category (Reference, Cat 1, Cat 2, Cat 3), averages and the lower and upper limits of the dose are specified. Odds ratio OR with 95 % confidence intervals (95 % CI) and the number of persons observed and the number of lung cancer deaths per dose category are reported

## Methods

### Reproduction of the results and variation of modelling

The complex meta-analysis of Vermeulen et al. [1] of the categorised results of the individual studies was replicated. The exposure categorisation, which varied with the studies, and the potential differences in the design and size of the studies were a challenge. In addition to this, the results for the exposure categories are nested within the studies, creating a two-level structure (1st level: exposure groups, 2nd level: studies). In order to analyse this complex data situation appropriately, meta-regression analyses were performed on the results of the individual studies, both with fixed effects [18] and with random effects [19–21], to determine a shared exposure-response curve (see also [22]).

The analyses must be weighted by variances of the RR estimates per exposure category. This weighting is required as only aggregated data (i.e., no individual data on persons) are available in the evaluations, and the data points for the exposure categories differ substantially in a joint evaluation due to the varying number of cases within and between the studies. Different approaches were trialled for this:

- Linear regression with fixed effects for log RR with weights proportional to the inverse of the respective variance
  - ◦ Without adjustment by study
  - ◦ With adjustment by study. A global F-test on the heterogeneity of the risk levels (intercepts, offsets) between the studies was performed.
- Mixed linear regression for log RR with a random intercept incorporating the differences between the studies
  - ◦ With weights at the first level (exposure categories level) proportional to the inverse of the respective variance and with the totals of these weights as study weights at the second level (study level)
  - ◦ In a second evaluation approach, the weights of the first level were scaled effectively to the weights of the second level.
- Mixed linear regression for log RR with a random intercept and a random dose coefficient (slope) incorporating the differences between the studies
  - ◦ With weights at the first level (exposure categories level) proportional to the inverse of the respective variance and with the totals of these weights as study weights at the second level (study level)
  - ◦ In a second evaluation approach, the weights of the first level were scaled effectively to the weights of the second level.

**Fig. 1:**
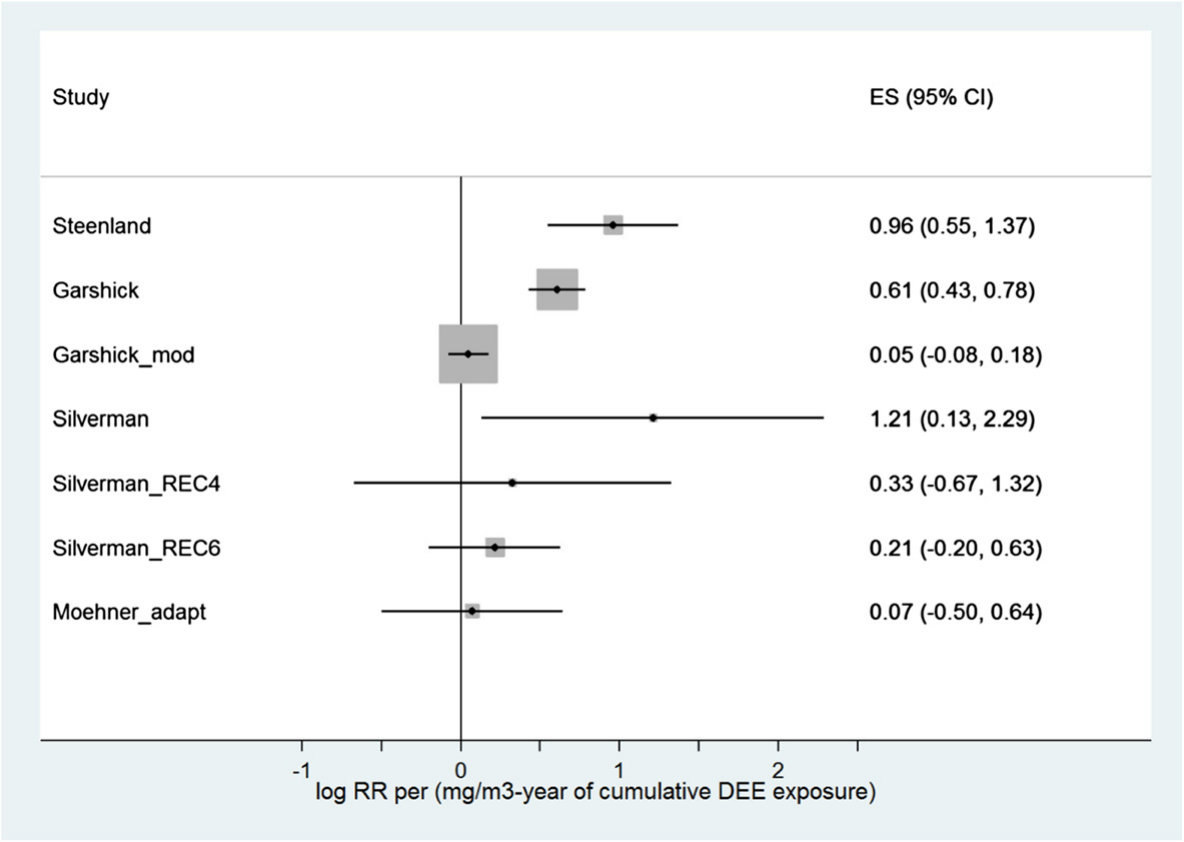
Analysis of the individual studies. Linear regression with fixed effects for log RR with weights proportional to the inverse of the respective variance (represented by box size). Coefficients (effect size, ES) of the cumulative exposure (Dose) to DEE in mg/m^3^-years with a 95 % confidence interval for the coefficient, calculated using the standard normal distribution. Steenland et al. [2] and Silverman et al. [4] were evaluated as used by Vermeulen et al. [1] as well as in accordance with the re-analysis by Crump et al. [14] with adjustment for the radon exposure (REC4, REC6, cf. Table 3), as was Garshick et al. [3] as used by Vermeulen et al. [1], and Garshick et al. [3] (modified) and the data based on Möhner et al. [17] (adapted)

The analyses do not take the reference points into account, as their weights are infinite, i.e., not a real number. That corresponds to the procedure in Vermeulen et al. [1]: Fig. 1 only contains 10 observation points, not 13.

Models with fixed effects are usually evaluated statistically via the Student’s t-distribution [23]. As only a few data points are incorporated, which reflect groups rather than individuals (aggregated data), this tends to lead to over-estimations of P values and confidence interval widths. However, precision weightings only include the relative weighting differences between the data points in the analyses and, thus, does not solve this problem. Therefore, the models with fixed effects are evaluated based on the standard normal distribution as an alternative [23]. This tends to lead to P values and confidence intervals which are too narrow, in particular as the correlations between the groups within the studies are not taken into consideration. We note that all mixed regressions are always evaluated via the standard normal distribution.

However, the results reported in a study for the various exposure categories are not independent of one another, but correlate with one another, as they refer to a common reference category within this study. As a result, in addition to the evaluations described above, meta-regression methods including the correlations of the study results within the studies were attempted [24–26]. However, additional input is required to use this Greenland/Longnecker method: person years and case numbers or person numbers and case numbers per exposure category from the studies must be available. These data are not included in the supplement to Vermeulen et al. [1], and cannot be reconstructed in full from the original publications.

In their Methods section, Vermeulen et al. [1] mentioned almost all of these methods, but it remains unclear whether they always used the Greenland/Longnecker method, for instance, and which weighting structure was used in the mixed regressions. With different evaluation approaches, this research project aims to evaluate the extent to which and the method with which the results published by Vermeulen et al. [1] can be reproduced.

Vermeulen et al. [1] also used spline models, which permit a more flexible curve shape than log-linear models. However, these spline regressions did not result in a deviating curve for the estimated exposure-response relationship: “The linear model (Fig. 1) and the spline meta-regression model (data not shown) fit the data well, with virtually equivalent curves.” That is why we did not use any spline functions or similar methods, instead following the main approach of Vermeulen et al., also to keep the number of parameters to be estimated as low as possible.

As the main modelling approach, a precision-weighted regression analysis with fixed effects and simultaneous adjustment for the individual studies was pursued, as it is more stable and easier to interpret. This approach also has other advantages over a regression with random effects (see the justifications in Allison [18], pp. 2, 3, and Cameron and Trivedi [19], p. 700). We examined whether the other approaches outlined above offer relevantly different results. If that is not the case, this method is used as the leading analytical strategy.

All analyses were calculated using Stata 12 [27].

### Influence of the input data selected

One important aspect of data selection can be derived from the Letter to the Editor by Morfeld [13] regarding the Garshick et al. [3] paper, stating that the coefficients used by Vermeulen et al. [1] are incorrectly adjusted. Therefore, another analysis was performed to repeat the meta-analyses described above with the corrected coefficients per Garshick et al. [3] (modified) (cf. Table 2).

The results of the DEMS re-analysis were presented in an HEI (Health Effects Institute) webinar [28, 29] and published in detail afterwards [14, 30]. Crump et al. [14] contained revised OR estimates on Silverman et al. [4]; important alternative estimates from this paper are presented in Table 3. The “REC6” findings from this paper are incorporated in the meta-analysis after adjustment for the radon exposure instead of the data from Silverman et al. [4] (cf. Table 1). Variation in the results when using the “REC4” findings after adjustment for radon exposure was also investigated.

The German potash miner study [17] was also incorporated as part of a sensitivity analysis. We used the risk estimates from “Möhner et al. [17] (adapted)” (cf. Table 4).

As the paper by Steenland et al. [2] has significant limitations (see the “Material” section), meta-analyses were also performed without including this study.

### Non-linearity: threshold search

We examined the data for non-linearities in the exposure-risk relationship. In particular, a systematic search was performed for cumulative exposure thresholds [31–33]. To do so, the algorithm presented in detail in Morfeld et al. [34] to determine a no-adverse effect level in the cumulative exposure was combined with the meta-regression methods. This analysis was restricted to the main modelling approach (precision-weighted regression analysis with fixed effects and simultaneous adjustment for individual studies).

10 μg/m^3^-years was selected as the increment for threshold exploration (range: 0 μg/m^3^-years to 500 μg/m^3^-years). Accordingly, 51 models were calculated and compared per threshold search. The threshold analysis was made using special programs in Stata 12 [27].

## Results

### Reproduction of the results in Vermeulen et al. [1] and extended analyses

In Table 1 (primary analysis), Vermeulen et al. [1] reported on the three studies by Steenland et al. [2], Garshick et al. [3] and Silverman et al. [4]. The authors stated individual findings (risk coefficients calculated) and the result of the meta-analysis of the risk coefficients. We compare these findings with the results of the re-analysis below.

### Analyses of the individual studies

All individual analyses were recalculated with fixed effects linear regression evaluating log RR (for methodological reasons, this analysis is identical to the mixed regression analysis if confined to a single study). Tables 5, 6, 7, 8 show the results for Steenland et al. [2], Garshick et al. [3], Silverman et al. [4] and Möhner et al. [17].

**Table 5:** Steenland et al. [2]: Linear regression with fixed effects for log RR with weights proportional to the inverse of the respective variance

Log RR	Coef.	Std. Err.	t	*P*	95 % CI	
Dose	0.00096	0.00021	4.55	0.045 (<0.001)	0.00005 (0.00055	0.00187 0.00137)
Constant	−0.031	0.070	−0.45	0.70	−0.33	0.27

Coefficient (Coef.) of the cumulative exposure (dose) to DEE in μg/m^3^-years and absolute term (constant) with standard deviation (Std.Err.); t-test statistic for the coefficient with the corresponding P-value and a 95 % confidence interval for the coefficient, 95 % CI. The figures in parentheses are the results when using the standard normal distribution

**Table 6:** Garshick et al. [3]: Linear regression with fixed effects for log RR with weights proportional to the inverse of the respective variance

log RR	Coef.	Std. Err.	t	*P*	95 % CI	
Dose	0.00061	0.000091	6.6	0.095 (<0.001)	−0.00055 (0.00042	0.00177 0.00078)
Constant	0.244	0.013	18.6	0.034	0.078	0.411
Garshick (modified)						
Dose	0.00005	0.00007	0.75	0.59 (0.45)	−0.00077 (−0.00008	0.00087 0.00017)
Constant	0.159	0.009	16.87	0.038	0.039	0.279

Coefficient (Coef.) of the cumulative exposure (Dose) to DEE in μg/m^3^-years and absolute term (Constant) with standard deviation (Std.Err.); t-test statistic for the coefficient with the corresponding P-value and a 95 % confidence interval for the coefficient, 95 % CI. The figures in parentheses are the results when using the standard normal distribution. Garshick et al. [3] as used by Vermeulen et al. [1], and Garshick et al. [3] (modified) were evaluated

**Table 7:** Silverman et al. [4]: Linear regression with fixed effects for log RR with weights proportional to the inverse of the respective variance

log RR	Coef.	Std. Err.	t	*P*	95 % CI	
Dose	0.00121	0.00055	2.20	0.27 (0.028)	−0.00579 (0.00013	0.00821 0.00229)
Constant	−0.148	0.299	−0.49	0.71	−3.95	3.65
Crump et al. [14], REC4						
Dose	0.00033	0.00051	0.64	0.63 (0.53)	−0.00615 (−0.00067	0.00680 0.00132)
Constant	0.00541	0.358	0.02	0.99	−4.54	4.55
Crump et al. [14], REC6						
Dose	0.00021	0.00021	1.01	0.50 (0.31)	−0.00247 (−0.00020	0.00289 0.00063)
Constant	0.137	0.108	1.27	0.42	−1.23	1.51

Coefficient (Coef.) of the cumulative exposure (Dose) to DEE in μg/m^3^-years and absolute term (Constant) with standard deviation (Std.Err.); t-test statistic for the coefficient with the corresponding P-value and a 95 % confidence interval for the coefficient, 95 % CI. The figures in parentheses are the results when using the standard normal distribution. Silverman et al. [4] was evaluated as used by Vermeulen et al. [1], and in accordance with the re-analysis by Crump et al. [14] with adjustment for radon exposure (REC4, REC6, cf. Table 3)

**Table 8:** Möhner et al. [17] (adapted): Linear regression with fixed effects for log RR with weights proportional to the inverse of the respective variance

log RR	Coef.	Std. Err.	t	*P*	95 % CI	
Dose	0.00007	0.00029	0.24	0.85 (0.81)	−0.00364 (−0.00050	0.00378 0.00064)
Constant	0.119	0.492	0.24	0.85	−6.13	6.37

Coefficient (Coef.) of the cumulative exposure (Dose) to DEE in μg/m^3^-years and absolute term (Constant) with standard deviation (Std.Err.); t-test statistic for the coefficient with the corresponding P-value and a 95 % confidence interval for the coefficient, 95 % CI. The figures in parentheses are the results when using the standard normal distribution

Table 1 in Vermeulen et al. [1] reports the following coefficient estimates for Steenland et al. [2]: dose = 0.00096 (95 % CI: 0.00033, 0.00159), constant = −0.032. The agreement in the coefficients is very good. The confidence interval is consistent when both calculation methods are taken into account (Student’s t-distribution, normal distribution).

Table 1 in Vermeulen et al. [1] reported the following coefficient estimates for Garshick et al. [3]: dose = 0.00061 (95 % CI: −0.00088, 0.00210), constant = 0.24. This coefficient estimate is also very similar, and the interval estimate is a good match. The estimates differ clearly when we evaluate Garshick et al. [3] (modified): The dose coefficient is lower by more than an order of magnitude, and far from being significant (*P* > 0.4). A significant deviation of the constant (intercept) from the normal value zero is apparent. This normal value zero means a baseline level of the relative risk of 1, as is to be assumed for a DEE exposure of 0 μg/m^3^-years.

Table 1 in Vermeulen et al. [1] reported the following coefficient estimates for Silverman et al. [4]: dose = 0.00120 (95 % CI: 0.00053, 0.00187), constant = −0.18. The coefficient estimates here also agree well (there is probably a typing error in Vermeulen et al. [1]: −0.18 instead of −0.148). The interval estimates are compatible too.

By contrast, the dose coefficients calculated using the Crump variations are far lower than the original values: They are only 27 % (REC4) or 18 % (REC6) of the Silverman coefficient. In these calculations, the t-test confidence intervals are largely symmetrical around zero, so that neither model reveals a trend. Even using the standard normal distribution, there is no indication of a significant influence of the DEE exposure on lung cancer mortality when REC4 or REC6 are used as the exposure metrics. We like to emphasize that REC4 and REC6 are both adjusted for radon exposure as a potential confounder.

Table 8 shows the results obtained by evaluating the study by Möhner et al. [17] with changed (adapted) exposure categorisation. The German study does not indicate an association between DEE exposure and lung cancer mortality.

Figure 1 gives an overview of the results of the regressions with fixed effects, assuming a normal distribution for statistical evaluation of all individual studies. Models with fixed effects are presented to prevent underestimation of the coefficients. We selected the normal distribution to rule out overestimation of the p-values (i.e., the confidence intervals presented are definitely not too wide). Deviating from Vermeulen et al. [1], we selected mg/m^3^-years = 1000 × μg/m^3^-years as the exposure unit, to keep the overview clearer. Each of the three data sets incorporated in the Vermeulen analysis [2–4] results in significantly elevated risk estimates, whereby Garshick et al. [3] paper has the greatest influence. When we analysed the results from Garshick et al. [3] without adjusting for exposure duration, i.e., Garshick et al. [3] (modified), the risk estimate drops considerably and is not significant. The Crump modifications (REC4, REC6) of the research in Silverman et al. [4] also result in considerably lower risk estimates than in the original Silverman et al. paper, and are not statistically significant. The study by Möhner et al. [17] does not give any indication of an association between DEE exposure and lung cancer mortality.

### Joint analysis of the studies

According to Table 1 in Vermeulen et al. [1], the following values apply for “all studies combined” (i.e., [2–4]) as the coefficients of cumulative exposure (dose) to DEE in μg/m^3^-years and the absolute term (constant): dose = 0.00098 (95 % CI: 0.00055, 0.00141) and constant = 0.088. Table 9 reports on the re-analysis of the three studies using a log-linear regression with fixed effects, without adjusting for the studies.

**Table 9:** Linear regression with fixed effects for log RR with weighs proportional to the inverse of the respective variance, without adjustment for studies

log RR	Coef.	Std. Err.	t	*P*	95 % CI	
Dose	0.00076	0.00026	2.97	0.018 (0.003)	0.00017 (0.00026	0.00135 0.00126)
Constant	0.130	0.072	1.80	0.11	−0.0365	0.297
Crump et al. [14], REC4						
Dose	0.00033	0.00022	1.46	0.18 (0.15)	−0.00019 (−0.00011	0.00084 0.00076)
Constant	0.208	0.067	3.10	0.015	0.053	0.336
Crump et al. [14], REC6						
Dose	0.00034	0.00020	1.74	0.12 (0.083)	−0.00011 (−0.00004	0.00080 0.00073)
Constant	0.211	0.054	3.90	0.005	0.860	0.335

Coefficient (Coef.) of the cumulative exposure (dose) to DEE in μg/m^3^-years and absolute term (Constant) with standard deviation (Std.Err.); t-test statistic for the coefficient with the corresponding P-value and a 95 % confidence interval for the coefficient, 95 % CI. The figures in parentheses are the results when using the standard normal distribution. Silverman et al. [4] was incorporated as used by Vermeulen et al. [1], and in accordance with the re-analysis by Crump et al. [14] with adjustment for radon exposure (REC4, REC6, cf. Table 3)

Table 9 shows relevant deviations from the results in Vermeulen et al. [1]. While the exposure-response association is statistically significant, the dose coefficient estimated without adjustment for the studies is approx. 20 % lower than that published by Vermeulen and colleagues (0.00076/0.00098 = 0.77). If the versions of the results according to Crump et al. [14] are incorporated in the evaluation, the meta-analysis does not indicate a significant association between DEE exposure and lung cancer mortality.

Table 10 also adjusts the coefficient estimates by studies.

**Table 10:** Linear regression with fixed effects for log RR with weights proportional to the inverse of the respective variance, with adjustment for studies

log RR	Coef.	Std. Err.	t	*P*	95 % CI	
Dose	0.00106	0.00020	5.35	0.002 (<0.001)	0.00057 (0.00067	0.00154 0.00144)
ΔConst1	0.249	0.080	3.11	0.021	0.052	0.445
ΔConst2	−0.035	0.126	−0.28	0.79	−0.343	0.272
Constant	−0.059	0.079	−0.74	0.49	−0.252	0.135
Crump et al. [14], REC4						
Dose	0.00053	0.00021	2.49	0.047 (0.013)	0.00001 (0.00011	0.00105 0.00094)
ΔConst1	0.163	0.095	1.71	0.14	−0.070	0.396
ΔConst2	−0.167	0.169	−0.98	0.36	−0.581	0.248
Constant	0.091	0.091	1.01	0.35	−0.130	0.313
Crump et al. [14], REC6						
Dose	0.00054	0.00017	3.12	0.021 (0.0018)	0.00012 (0.00020	0.00096 0.00087)
ΔConst1	0.164	0.069	2.40	0.054	−0.003	0.332
ΔConst2	0.041	0.113	−0.37	0.73	−0.319	0.236
Constant	0.088	0.068	1.30	0.24	−0.079	0.255

Coefficient (Coef.) of the cumulative exposure (Dose) to DEE in μg/m^3^-years and absolute term (Constant) with standard deviation (Std.Err.); t-test statistic for the coefficient with the corresponding P-value and a 95 % confidence interval for the coefficient, 95 % CI. The figures in parentheses are the results when using the standard normal distribution. Silverman et al. [4] was incorporated as used by Vermeulen et al. [1], and in accordance with the re-analysis by Crump et al. [14] with adjustment for radon exposure (REC4, REC6, cf. Table 3)

Constant: Steenland et al. [2] (reference study)

ΔConst1: Garshick et al. [3] - Steenland et al. [2]

ΔConst2: Silverman et al. [4] - Steenland et al. [2]

Table 10 shows a good correlation for the dose coefficient and the corresponding confidence interval with the results reported in Vermeulen et al. [1] (dose = 0.00098, 95 %-CI: 0.00055, 0.00141; constant = 0.088). The recalculated coefficient exhibits minimal upward deviation. The two confidence interval calculations (Student’s t-distribution, normal distribution) have largely matching results, so that the 10 data points are sufficient to permit a robust estimate, which also agrees to the results published by Vermeulen and his colleagues. The global F-test on the heterogeneity of the baseline risk levels between the studies, which was also calculated, results in F(2, 6) = 5.5, *P* = 0.044. Thus, the heterogeneity between the three studies is significant at a 5 % level. Heterogeneity refers to a systematic difference in the baseline risk of the three studies (i.e., setting exposure to zero). For a meaningful combination of the three studies, their base levels should match except for random deviations. However, the differences are statistically significant. The heterogeneity between the three studies results from a significant difference between Garshick et al. [3] and Steenland et al. [2], ΔConst1: p = 0.021. Accordingly, the Garshick study deviates significantly upwards in the baseline level of the risk from the other two studies, as was already indicated in the individual analysis (Table 6). Correcting the Garshick coefficients reduces the heterogeneity in the risk level considerably: F(2, 6) = 0.50, *P* = 0.63. This is not apparent from the individual analysis.

If we replace the original data per Silverman et al. [4] with the results from Crump et al. [14] (REC4 or REC6, both adjusted for radon exposure), the dose coefficients in the meta-regression are halved.

Table 11 shows results when a mixed linear regression for the three studies is fitted.

**Table 11:** Mixed linear regression for log RR with a random intercept with weights at the first level (exposure categories level) proportional to the inverse of the respective variance and with the totals of these weights as study weights at the second level (study level)

log RR	Coef.	Robust Std. Err.	Z	*P*	95 % CI	
Dose	0.00097	0.00014	6.74	<0.001	0.00069	0.00125
Constant	0.084	0.111	0.75	0.45	−0.134	0.300
Garshick (modified)						
Dose	0.00087	0.00019	4.58	<0.001	0.00050	0.00124
Constant	0.024	0.062	0.39	0.70	−0.097	0.145
Crump et al. [14], REC4						
Dose	0.00045	0.00021	2.10	0.035	0.00003	0.00086
Constant	0.178	0.094	1.90	0.057	−0.005	0.362
Crump et al. [14], REC4 and Garshick (modified)						
Dose	0.00048	0.00023	2.10	0.036	0.00003	0.00092
Constant	0.097	0.040	2.39	0.017	0.018	0.176
Crump et al. [14], REC6						
Dose	0.00053	0.00026	2.05	0.040	0.00002	0.00103
Constant	0.172	0.101	1.71	0.088	−0.025	0.369
Crump et al. [14], REC6 and Garshick (modified)						
Dose	0.00052	0.00025	2.09	0.036	0.00003	0.00101
Constant	0.095	0.051	1.87	0.061	−0.044	0.194

The weights of the first level are scaled effectively to the weights of the second level. Coefficient (Coef.) of the cumulative exposure (Dose) to DEE in μg/m^3^-years and absolute term (Constant) with standard deviation (Std.Err.); Z-test statistic for the coefficient with the corresponding P-value and a 95 % confidence interval for the coefficient, 95 % CI. Silverman et al. [4] was incorporated as used by Vermeulen et al. [1], and in accordance to the re-analysis by Crump et al. [14] with adjustment for the radon exposure (REC4, REC6, cf. Table 3). Garshick et al. [3] as used by Vermeulen et al. [1], and Garshick et al. [3] (modified) were incorporated

Table 11 results in a very good agreement to the finding published in Vermeulen et al. [1] (dose = 0.00098, 95 % CI: 0.00055, 0.00141; constant = 0.088). If the dose coefficient is also estimated as a random effect, the results do not change. The findings deviate slightly from Vermeulen et al. [1] without an effective scaling of the weights (result: dose coefficient = 0.00093, constant =0.096). If Garshick et al. [3] (modified) is included in the meta-analysis, the estimated meta-risk coefficient and the corresponding significance level decreases (test statistic changes from Z = 6.74 to Z = 4.58), however the positive dose–response association is always significant (see Table 11).

As in the models with fixed effects (Table 10), the dose coefficients in the meta-regression are halved in the mixed regression (Table 11) if the original data in accordance with Silverman et al. [4] is replaced with the findings from Crump et al. [14] (REC4 or REC6, both adjusted for radon exposure).

Crump [39] also recalculated some of the Vermeulen et al. [1] results using a mixed model, but did not see any way to obtain the data required to use the Greenland/Longnecker method (cf. our explanations in the Method section “Reproduction of the results and variation of modelling”). Crump wrote: “I … reran the analysis of Vermeulen et al. [1], except that I did not model the dependence among the ORs from the same study. (I did not have access to data needed to model that dependence.) My analysis yielded a regression parameter [0.88; 95 % confidence interval (CI): 0.65, 1.11] similar to that obtained by Vermeulen et al. [1] (0.98; 95 % CI: 0.55, 1.41).“ Crump [29] obviously selected a different unit of DEE exposure, at 1000 μg/m^3^-years. The dose coefficient (0.88) he reported deviates more than the result of the recalculation performed in this paper (0.97), which corresponds very well with the Vermeulen result (0.98). It is not clear whether Crump scaled the weights, as even the recalculation in this report is slightly lower (0.93) without scaling, though not as pronounced as in Crump [29].

### Variation of modelling

As Steenland et al. [2] did not provide details on the person years and case numbers per exposure category, this paper cannot be included in evaluations with the Greenland/Longnecker method. However, Garshick et al. [3], Silverman et al. [4] and Möhner et al. [17] (adapted) can be analysed jointly. Table 12 shows the results.

**Table 12:** Joint analysis of Garshick et al. [3] (modified coefficients), Silverman et al. [4] and Möhner et al. [17] (adapted)

	Coef.	*P*	95 % CI	
Fixed effects, adjusted	0.00054	0.14 (0.083)	−0.00026 (−0.00007	0.00133 0.00115)
Random intercept, scaled	0.00024	0.36	−0.00027	0.00074
Greenland/Longnecker method	0.00032	0.035	0.00002	0.00062
Crump et al. [14], REC4				
Fixed effects, adjusted	0.00020	0.35 (0.30)	−0.00029 (−0.00018	0.00069 0.00058)
Random intercept, scaled	0.00010	0.11	−0.00002	0.00022
Greenland/Longnecker method	0.00015	0.31	−0.00014	0.00044
Crump et al. [14], REC6				
Fixed effects, adjusted	0.00013	0.33 (0.28)	−0.00018 (−0.00010	0.00042 0.00035)
Random intercept, scaled	0.000073	0.025	0.00001	0.00014
Greenland/Longnecker method	0.00012	0.44	−0.00019	0.00043

Comparison of the estimated effect for the cumulative exposure to log RR, calculated with various regression methods. Coefficient (Coef.) of the cumulative exposure (Dose) to DEE in μg/m^3^-years, corresponding P-value and 95 % confidence interval for the coefficient, 95 % CI. The figures in parentheses are the results when using the standard normal distribution in the model with fixed effects. Silverman et al. [4] was incorporated as used by Vermeulen et al. [1], and in accordance with the re-analysis by Crump et al. [14] with adjustment for radon exposure (REC4, REC6, cf. Table 3)

Table 12 shows that the linear regressions with fixed effects for log RR with weights proportional to the inverse of the respective variance and with adjustment for studies result in slightly more pronounced effect coefficients and somewhat lower P-values than models with random effects (that qualitatively matches the findings presented in the Results section: “Joint analysis of the studies”). However, if the dose coefficient is also calculated as a random effect, a similar value results (coefficient = 0.00059, P = 0.17) for the model with fixed effects. By contrast, the analyses with the Greenland/Longnecker method, recommended by methodologists and taking internal correlations into account, resulted in a lower coefficient than in the model with fixed effects, with a lower P-value = 0.035 (significant). In this joint analysis of Garshick et al. [3] (modified coefficients), Silverman et al. [4] and Möhner et al. [17] (adapted) using the Greenland/Longnecker method, the dose coefficient is approximately a factor of 3 lower than the estimate in the primary analysis by Vermeulen et al. [1] (0.0032 vs. 0.00098).

The results reported by Vermeulen et al. [1] largely match the estimates in the models with random effects (cf. Results section on the “Joint analysis of the studies”). However, the Greenland/Longnecker method cannot be used in this case without access to further data. Vermeulen and his colleagues were obviously given access to these data from Steenland et al. [2], but did not disclose them in their paper, even in the supplement.

If we replace the original data per Silverman et al. [4] with the results from Crump et al. [14] (REC4 or REC6, after adjustment for radon exposure), the statements on the three analysis methods are qualitatively the same. Accordingly, the dose coefficients in the meta-regression are halved due to the data variation. In spite of the different models, the P-values are largely equivalent. One exception to this is regression with a random intercept for REC6, where the value is lower (P = 0.25). Like the deviating result of the Greenland/Longnecker regression on evaluation with the Silverman original data (P = 0.035), this indicates instabilities in the variance estimate.

In order to avoid discussions on a potential underestimation of the effect, all further analyses were made using adjusted regression models with fixed effects. In Tables 11 and 12, the dose coefficient estimates (fixed effects adjusted for studies and random effects) almost match, and are also almost identical with those in Vermeulen et al. [1]. A comparison of the interval estimates and P-values indicates a greater stability of the regression models with fixed effects. This justifies the decision to use adjusted regression models with fixed effects as a primary analysis method (main modelling approach) for this research project.

### Influence of the input data selected

Regardless of the method selected, a simultaneous analysis of the three studies by Garshick et al. [3] (modified), Silverman et al. [4] and Möhner et al. [17] (adapted) results in a far lower effect coefficient in Table 12 than in the primary analysis by Vermeulen et al. [1]. Example: For the regression models with fixed effects and adjustment for studies, a reduction in the effect coefficient by approx. 50 % resulted, as the reproduced primary analysis in accordance with Vermeulen et al. [1] led to a coefficient of 0.0011 (per 1 μg/m^3^-years), P = 0.002, and a 95 % CI of 0.00057 to 0.00154 (cf. Table 10).

An interesting finding to be noted is that a simultaneous analysis of the three studies by Garshick et al. [3] (modified), Silverman et al. 2012 and Möhner et al. [17] (adapted) only finds a significant dose–response relationship (Table 12) if the Greenland/Longnecker method is used. This could be due to instabilities in estimation in this complex procedure.

If the primary analysis in accordance with Vermeulen et al. [1] is performed with fixed effects and adjustment for studies and with modified Garshick coefficients, this results in a coefficient of 0.00098 (per 1 μg/m^3^-year), *P* = 0.006, and a 95 % CI of 0.00041 to 0.00155, somewhat more pronounced but similar to the results of the regression calculation with random effects (cf. Table 11).

Thus, the modification of the Garshick coefficients only reduces the meta-coefficient by approx. 7 % and the statistical significance is maintained, although the P-value increases from 0.002 to 0.006. Correcting the Garshick coefficients reduces the heterogeneity in the base level of the risk considerably: F(2, 6) = 0.50, *P* = 0.63. Without correction we get: F(2, 6) = 5.5, *P* = 0.044.

Tables 10, 11, 12 are consistent in showing that replacing the original data from Silverman et al. [4] with the results from Crump et al. [14] (REC4 or REC6, both with adjustment for radon) reduces the dose coefficient by approximately half in the meta-regressions. If we analyse Steenland et al. [2], Crump et al. [14] and Garshick et al. [3] (modified) together, the dose–response relationship in adjusted regression models with fixed effects and evaluation with the standard normal distribution is significant (REC4: *P* = 0.03, REC6: *P* = 0.01), but clearly weaker than when analysing the original data used by Vermeulen et al. [1] (*P* < 0.000001). The lower confidence interval limits of the dose coefficient are correspondingly different: 0.00005, 0.00010 and 0.00067. They differ by a factor of at least 12 or 6.

**Fig. 2:**
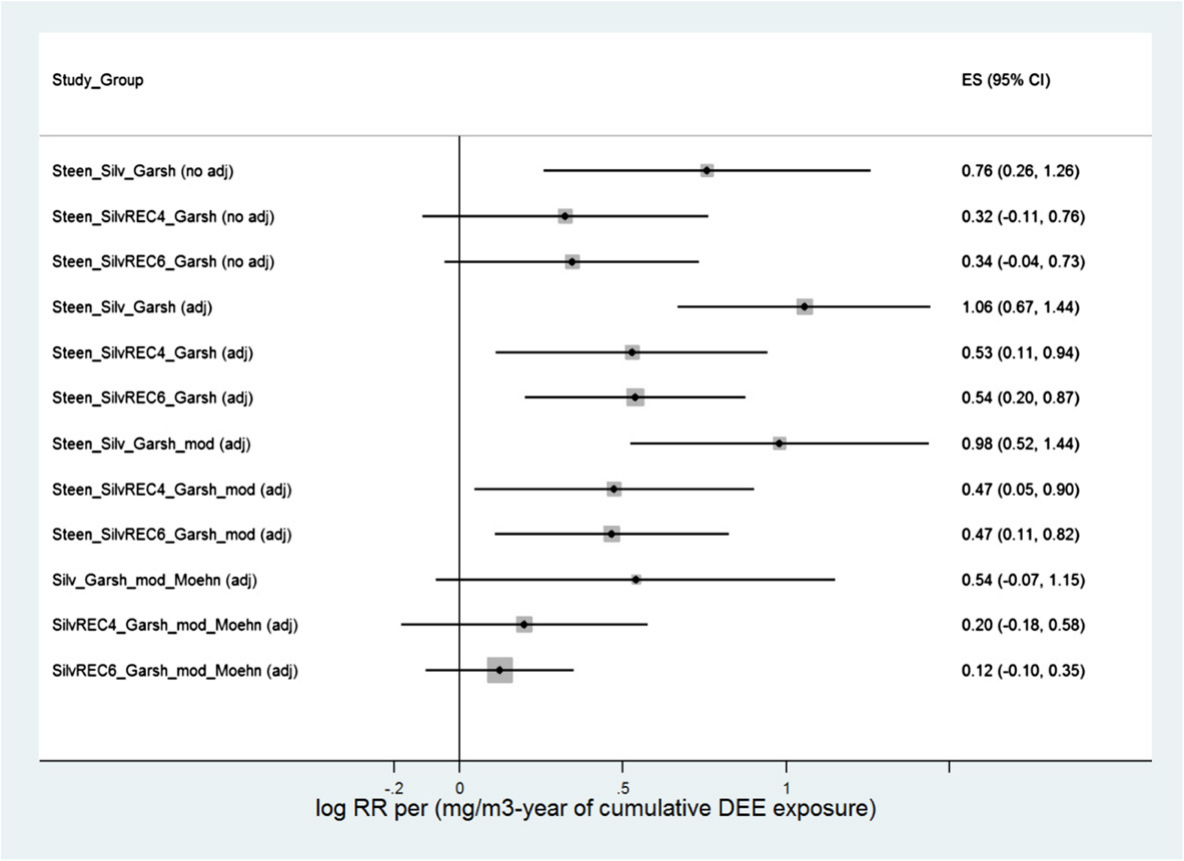
Meta-analyses. Linear regression with fixed effects for log RR with weights proportional to the inverse of the respective variance (the box size illustrates the precision of the meta-estimate). Coefficients (effect size, ES) of the cumulative exposure (Dose) to DEE in mg/m^3^-years with a 95 % confidence interval of the coefficient, calculated using the standard normal distribution. Steenland et al. [2] and Silverman et al. [4] were evaluated as used by Vermeulen et al. [1], as well as in accordance with the re-analysis by Crump et al. [14] with adjustment for the radon exposure (REC4, REC6, cf. Table 3), as was Garshick et al. [3] as used by Vermeulen et al. [1], and Garshick et al. [3] (modified) and the data based on Möhner et al. [17] (adapted)

Figure 2 gives an overview of the results of the meta-regressions with fixed effects for variations of the input data assuming a normal distribution for statistical evaluation. Models with fixed effects are presented to prevent underestimation of the coefficients. We selected the normal distribution to rule out overestimation of the p-values (i.e. the confidence intervals presented are definitely not too wide). Deviating from Vermeulen et al. [1], we selected mg/m^3^-years = 1000 × μg/m^3^-years as the exposure unit, to keep the overview clearer. Replicating the analysis as performed in Vermeulen et al. [1], i.e. Steen_Silv_Garsh (adj), results in the highest risk estimate of all data variations. If the modifications of the Silverman case–control study in accordance with Crump et al. 2015 (REC4, REC6, both adjusted for radon exposure) and Garshick et al. [3] are included in the analysis, the risk estimates are far lower (Steen_SilvREC4_Garsh_mod, Steen_SilvREC6_Garsh_mod: the coefficients are reduced roughly by half compared with the Vermeulen analysis). If the Steenland paper, which the authors believe is only exploratory in nature, is excluded, the modified risk estimates do not result in significant risk increases when incorporating Möhner et al. [17] (adapted) in the meta-analysis (SilvREC4_Garsh_mod_Moehn, SilvREC6_Garsh_mod_Moehn). The meta-coefficient is approx. 10 – 20 % of the value found in Vermeulen et al. [1] in the primary analysis.

### Non-linearity: Threshold search

The primary analysis by Vermeulen et al. [1] was repeated (method: adjusted regression models, fixed effects), but taking a potential threshold into consideration. When evaluated with corrected coefficients in Garshick coefficients (cf. Table 2: Garshick et al. [3], modified), the analysis finds a threshold of 150 μg/m^3^-years. However, the threshold does not differ statistically significantly from zero.

If we replace Silverman et al. [4] (Table 1) with the results from Crump et al. [14] (Table 4), the analysis also exhibits a threshold. However, this estimate is more difficult to express statistically due to the considerably weaker exposure-response relationship, and exhibits a broad uncertainty (threshold at 90 μg/m^3^-years, 95 % CI: 0 μg/m^3^-years to 361 μg/m^3^-years).

## Discussion

Vermeulen et al. [1] published a meta-regression analysis of three major epidemiological US studies [2–4] which analyzed the association between diesel engine exhaust (DEE), based on the cumulative exposure to elemental carbon (EC) in μg/m^3^-years, and lung cancer mortality. In their meta-analysis, the authors described a statistically significant dose–response relationship and elevated cancer risks, even at very low exposures. The present re-analysis largely succeeded in reproducing the individual and main findings from the published study data. Of all the meta-analyses we performed, the evaluation of the data as used by Vermeulen et al. [1] resulted in the highest risk estimates. However, an investigation of the heterogeneity in the baseline level of risk – Vermeulen et al. [1] do not report on this – resulted in pronounced differences between the three studies (significant at the 5 % level). All three studies should exhibit a uniform baseline level of RR = 1 at a cumulative DEE exposure of 0 μg/m^3^-years. A joint analysis of the three studies must therefore be viewed critically from a statistical point of view [34]. Other authors in other situations reject combinations of studies even with considerably less pronounced heterogeneity [35, 36]. This uncertainty in the baseline level renders the use of the analysis by Vermeulen et al. [1] a problem for risk estimates in the lower exposure region. Accordingly, the following statement by Vermeulen et al. [1] must be relativised: “Formal tests of heterogeneity of estimates among the studies were of limited value due to the small number of data points for each study.”

Correcting the coefficients in Garshick et al. [3] [cf. 13] reduced the heterogeneity in the baseline relative risk levels between the three studies considerably (*P* = 0.63). This correction was indicated as the coefficients used by Vermeulen et al. [1] are incorrectly adjusted [13]: The cumulative exposure was adjusted for the exposure duration it already contains so that the risk coefficient does not estimate the effect of cumulative exposure. Our modification resulted in far lower risk estimates than reported on this study by Vermeulen et al. [1]: The corrected dose coefficient is lower by more than an order of magnitude, and far from being significant (*P* = 0.6). However, the correction of the Garshick coefficients only reduces the meta-coefficient by approx. 7 % and the statistical significance is maintained, although the P-value increases from 0.002 to 0.006.

Another aspect is important for correct evaluation of the Garshick study (see the Letter to the Editor by Morfeld [13], including the answer from the authors). Garshick et al. [3] made a double adjustment for the year of birth, though with different fineness, so that the models do not break down due to collinearity. However, over-adjustments of this type can distort the coefficient estimate. The authors responded that they incorporated the years twice to obtain meaningful results. That was the only way the proportional hazards assumption was fulfilled. However, this explanation does not change the double use of the year of birth information, and the potential over-adjustment it causes.

The highest exposure value included in the meta-study came from the DEMS case–control study by Silverman et al. [4]: 1036 μg/m^3^-years with an OR = 2.83 (95 % CI: 1.28 to 6.26). Thus, the US mining study is particularly relevant for the meta-analysis. Critical comments and a list of open questions were published [37] on the DEMS publications [4, 12], to which the authors reacted with a letter to the editor [38]. However, many aspects remain open [see the author answer from Morfeld in 38]. Some of these open questions could be answered by additional analyses, which requires access to the original data. Access to the original data of the DEMS study has only been granted to a few researchers (working groups of S. Moolgavkar [30] and K. Crump [14]). It is currently unclear whether these authors had unrestricted access to all original data. However, aspects of the manner in which the Crump et al. [14] team was given access to the DEMS data is provided in the “Epilogue” section of their paper. The results of the DEMS re-analysis were presented in a HEI (Health Effects Institute) webinar [28, 29] and published in detail recently [14, 30].

In a Letter to the Editor on Vermeulen et al. [1], Crump [39] reported that an evaluation of the DEMS case–control study with an exposure lag of 5 years leads to far lower meta-risk coefficients than with the original value of 15 years. Crump also pointed out that the other two studies incorporated [2, 3] used a lag of 5 years. The authors qualitatively confirmed the Crump result, but did not consider it relevant, as the adjustment in the DEMS study using a lag of 15 years was better and there are inevitably differences in the exposures recorded in the different studies. Furthermore, Vermeulen et al. [1] referred to a sensitivity analyses they performed, in which they explored the influence a different lagging of the exposure has. However, the note by Crump [39] shows further uncertainties in the Vermeulen meta-analysis, which cannot be determined without access to the original DEMS data. Our re-analysis should be repeated applying different lags to the studies. Unfortunately, the necessary data are not publicly available.

Moolgavkar et al. [30] re-analysed, using both proportional hazard and biological based mechanistic models, the DEMS cohort study by Attfield et al. [12] and pointed out two important aspects:

1) Time-dependent factors are superimposed, so that model coefficients should not be estimated without considering the interaction with age. Accordingly, stating an isolated risk coefficient – as described in Attfield et al. [12] – does not make any sense according to Moolgavkar et al. [30].

2) One mine (limestone mine) is an outlier in the data. The DEE exposures in this mine are the lowest, but the risk estimates are the highest of all the mines. A Cox regression analysis of the data (without excluding the higher exposures as in Attfield et al. [12]) shows that a significant exposure-response association (P = 0.0014) was found in the limestone mine alone. If the limestone mine is excluded from the analyses, the P-value in the overall cohort increases from 0.02 to 0.18 [28], i.e., after excluding the limestone mine, no significant dose–response association results between the DEE exposure and lung cancer mortality in the DEMS cohort study. Although this special role of the limestone mine was not confirmed in the case–control re-analysis by Crump et al. [14], this observation about the limestone mine is a major interpretation problem for the cohort study and the publication by Attfield et al. [12]. It is noteworthy that Moolgavkar et al. [30] described the ventilation in the limestone mine as follows: “All seven of these mines had substantial mechanical ventilation supplying large quantities of air to minimize airborne dust concentrations and, in the case of the trona mines, to minimize the build-up of methane, an explosive gas. The limestone mining operation was quite different from that in the seven other mines. The limestone mine primarily used natural ventilation, with air flowing up or down vertical shafts between the surface and the mining operations.” In addition, the percentage of radon measurements above detection limit was 85 % in the limestone mine, the highest among all 8 mines; the average percentage among the other 7 mines was only 36 % (Roger O. McClellen, personal communication, 27 July 2015). This may explain why Crump and colleagues were unable to reproduce Moolgavkar’s finding of the limestone mine being an outlier: “Moolgavkar et al. could not control for other covariables, including smoking and radon, because these data were not available” (Crump et al. [14]). Such a control for other covariables was done by Crump et al. [14].

Crump et al. [14] re-analysed the DEMS case–control study by Silverman et al. [4] and also investigated the influence of some covariables, which Silverman et al. [4] did not include in their final models. In the original paper, analysing the association of cumulative DEE exposure (lag = 15 years) and lung cancer mortality led to a trend *P*-value of *P* = 0.001 (Table 3, Silverman et al. [4]), which Crump et al. confirmed: *P* = 0.0006 in their re-analysis. Crump et al. [14] (Table 3) reported a trend P-value of P = 0.02, if adjustments are also made for radon exposure. This proves that the radon exposure has a considerable influence on the association, and weakens the statistical significance of the DEE variables after taking this covariable into account. Crump et al. [14] also developed six new DEE exposure metrics, as an alternative to the estimates used in Attfield et al. [12] and Silverman et al. [4]. If these alternative DEE exposure metrics are used, and also adjusted for the radon exposure, there is no significant association between the cumulative DEE exposure and lung cancer mortality in any constellation studied (*P* ≥ 0.17 or the trend is negative). The analyses by Crump et al. [14] also did not reveal any significant association for the exposure metrics as used in Attfield et al. [12] and Silverman et al. [4], when the individual data in the analysis were used instead of the average values of the groups, and an adjustment was made for radon exposure: *P* ≥ 0.65 or the trend is negative. The authors wrote: “Most importantly, we used the radon concentration data for the DEMS cohort provided by the DEMS investigators. When adjustment was made for radon, a known human lung carcinogen, the effect of REC on the association with lung cancer mortality was confined to only the three DEMS REC estimates. Most notably, there was no evidence of an association with any of the six alternate REC estimates, including REC6. When T2 trend tests were conducted, based on the use of individual worker REC estimates, the results were less statistically significant and in many cases the trends were negative. Indeed, for miners who always worked underground, five of the six REC metrics exhibited negative trends.”

Moolgavkar et al. [30] and Crump et al. [14] concluded that the DEMS analyses by Attfield et al. [12] and Silverman et al. [4] are not suitable for use on their own in a quantitative risk analysis. The limitations of this DEMS data must also be taken into consideration for meta-analyses and derived limit values. The issue of Risk Analysis that contained the Moolgavkar et al. [30] and Crump et al. [14] papers contained a “note” by the Editors, Cox and Lowrie, on the use of results of analyses of the same data set using alternative models [40]. The Editors described that the reanalysed data set was influential in IARC’s decision to classify DEE as a human carcinogen and concluded: “These findings can be viewed as raising important questions about the usefulness and reliability of expert judgements about the causal interpretation of model-dependent associations in general, and about whether DEE is in fact carcinogenic to humans in these studies in particular.”

If the paper by Crump et al. [14] (REC4, REC6, both adjusted for radon exposure) is incorporated instead of Silverman et al. [4], our meta-re-analysis of Vermeulen et al. [1] results in a considerably weakened association between DEE exposure and lung cancer mortality. Tables 10, 11, 12 are consistent in showing that a corresponding replacement of the original data roughly halves the dose coefficient in the meta-regression. If we analyse Steenland et al. [2], Crump et al. [14] and Garshick et al. [3] (modified) together, the dose–response association in an adjusted regression model with fixed effects is just about significant (*P* = 0.03), i.e., less certain than in the original data analysis (*P* < 0.000001).

We were unable to apply the recommended Greenland/Longnecker method on the oldest study, that of Steenland et al. [2], as the publication lacks important additional information. However, we did perform meta-analyses of Garshick et al. [3], Silverman et al. [4] and Möhner et al. [17] with this statistical method. Regardless of the evaluation method (random, fixed, Greenland/Longnecker), a joint analysis of Garshick et al. [3] (modified), Silverman et al. [4] and Möhner et al. [17] (adapted) resulted in similar coefficient estimates, whereby the Greenland/Longnecker estimate was between the results from the model with random effects and the adjusted model with fixed effects. This justified focussing our analyses on the adjusted model with fixed effects, which made analyses possible in all data situations. After excluding the explorative study by Steenland et al. [2], the lowest risk estimate resulted in a meta-analysis of the three studies Garshick et al. [3] (modified), Silverman et al. [4] (modified in accordance with Crump et al. [14] and Möhner et al. [17] (adapted). In this evaluation, the meta-coefficient decreased to approx. 10 % to 20 % of the value published by Vermeulen et al. [1] as a result of their primary analysis. In addition to this, the association between the DEE exposure and lung cancer mortality in this analysis is no longer statistically significant.

Vermeulen et al. [1] noted: “We were not able to investigate other model forms in our meta-regression, beyond the linear and spline curves because of the limited number of data points. If nonlinear exposure-response curves were actually a better fit (e.g., attenuation at higher exposures, for which there is some evidence in Silverman et al. (2012), then this might change the estimate burden of disease due to DEE.” If we supplement the meta-analysis of the three US studies [2–4]) with a threshold search, i.e., examine the dose–response relationship for non-linearity, and use the corrected Garshick coefficients it results in a threshold estimate for the cumulative DEE exposure at 150 μg/m^3^-years. However, this estimate does not differ significantly from zero.

Sun et al. [41] created an overview of the results from 42 cohort studies and 32 case–control studies on the association between the DEE exposures and lung cancer. The authors concluded: “Overall, neither cohort nor case–control studies indicate a clear exposure-response relationship between DE exposure and lung cancer. Epidemiological studies published to date do not allow a valid quantification of the association between DE and lung cancer.” Although this research does not reach the methodological level of the meta-analytical approach by Vermeulen et al. [1], the varying study results in Sun et al. [41] verify the uncertainty underlying epidemiological studies on the association between DEE and lung cancer.

Similarly to our analysis of the Vermeulen analysis, the re-analysis of the German potash miner study by Möhner et al. [17] led to a clearly weakened and different statement compared with the original research [42]: “Only for very high cumulative dose, corresponding to at least 20 years of exposure in the production area, some weak hints for a possible risk increase could be detected.”

The meta-regression analysis performed in this paper has considerable restrictions. Major limitations result from the fact that neither DEE concentration values (only details on cumulative exposures) nor the individual data for this research project were available. Threshold analyses for dust should focus on a concentration threshold [see the discussion in 43]. Empirical findings on thresholds in quartz dust exposure, based on the German porcelain worker cohort, did not result in a threshold for the cumulative exposure, but did for the concentration [43]. Similarly, it is probable in this case that the restriction of the data to cumulative DEE exposure means that we can assume that the actual concentration threshold is underestimated. The statistical significance of the finding would be far clearer if the original studies were analysed incorporating concentration values in the meta-analysis. The data are only available in aggregated form (grouped data) (cf. Table 1), while the original data are individual. Analyses of this type are difficult when using data already collapsed into categories, as results can occur depending on how the cut points are chosen, and precision of estimates decreases with categorisation in comparison to continuous analyses. In general, categorisations result in information losses, potential distortions and decreases in power [44].

The limitations mentioned affect the analysis by Vermeulen et al. [1] and our re-analysis accordingly. Although all main authors of the individual studies are co-authors of the meta-analysis, Vermeulen et al. [1] did not pool and analyse the original data, but focused on grouped data from the result tables of the three publications. However, reliable analyses should refer to the individual data and consider the DEE concentration as a key variable in analyses. We note that a similar point was made by Crump [39]: “Vermeulen et al. used very crude exposure summaries (e.g., midpoints of exposure intervals).”

The meta-regressions performed here show significant variations in the results, depending on the study data incorporated or the analytical methods applied. The data used by Vermeulen and colleagues led to the highest risk estimates in our meta-analysis (statistically significant). After excluding the explorative study by Steenland et al. [2], the lowest risk estimate resulted in an analysis of the three studies Garshick et al. [3] (modified), Silverman et al. [4] (modified in accordance with [14]) and Möhner et al. [17] (adapted). In this evaluation, the meta-coefficient decreased to approx. 10–20 % of the value published by Vermeulen et al. [1] as the main result of their primary analysis. The association between DEE exposure and lung cancer mortality in this analysis is not statistically significant. The risk estimates derived from Vermeulen et al. [1] in the very low exposure range and the corresponding limit value proposals derived [6] are therefore less than convincing, as the data – after correction of the Garshick coefficients – are also generally compatible with a threshold.

Toxicological results of the current ACES study from controlled long-term experiments on rats with new technology diesel exhaust (NTDE) did not exhibit tumour growth or precancerous conditions [45], by contrast to earlier studies with traditional DEE (TDE) from diesel motors without particle reduction and without other forms of exhaust treatment. The summary of IARC workshop results for re-evaluation of DEE in 2012 revealed that only toxicological data with pre-2000 motors and fuel technologies were incorporated [5]. Accordingly, the IARC classification from 2012 referred only to possible carcinogenic potential from diesel engine exhaust without modern exhaust treatment (TDE), using the epidemiological data from Steenland et al. [2], Garshick et al. [3], Silverman et al. [4]. However, the new results from the animal experiments in the ACES study highlight the need to include engine and exhaust treatment technologies in the discussion on workplace limit values and possible lung cancer risks from DEE. McClellan et al. [46] also explicitly mentioned the qualitative and quantitative differences between TDE and NTDE and recommended that these differences should be considered when evaluating the carcinogenic risks. The authors reviewed the substantial changes made in diesel technology, and the resulting changes in diesel exhaust emissions from post-WW II to the present time. The changes in technology and emissions post-1990 have been particularly dramatic. This raises questions with regard to the use of the findings of any of the epidemiological studies analysed in this paper for projecting lung cancer risks of diesel exhaust exposures post-2000.

Regardless of the fundamental restrictions mentioned above, the present re-analysis also revealed that the results of the meta-regression study by Vermeulen et al. [1] should not be used in any quantitative lung cancer risk evaluation without reservations, as the results vary significantly depending on the input data selected and the statistical methods used. This is particularly true for the low exposure region.

## Conclusions

- Vermeulen et al. [1] published a meta-regression analysis of three key epidemiological US studies on the association between diesel engine exhaust (DEE) and lung cancer. They found a statistically significant dose–response relationship and elevated cancer risks even for very low DEE exposures.
- The present re-analysis largely succeeded in reproducing the individual cohort results and main meta-findings of Vermeulen et al. [1] from the published study data. Our meta-regressions, however, show significant variations of the results, depending on the study data incorporated or the analytical methods applied.
- Therefore, the results of the meta-regression analysis by Vermeulen et al. [1] should not be used in a risk assessment without reservation, especially not in the low-DEE exposure range.
